# Palliative care team consultation and quality of death and dying in a university hospital: A secondary analysis of a prospective study

**DOI:** 10.1371/journal.pone.0201191

**Published:** 2018-08-23

**Authors:** Arianne Brinkman-Stoppelenburg, Frederika E. Witkamp, Lia van Zuylen, Carin C. D. van der Rijt, Agnes van der Heide

**Affiliations:** 1 Department of Public Health, Erasmus MC University Medical Center Rotterdam, Rotterdam, the Netherlands; 2 Faculty of Nursing and Center of Expertise in Care Innovations, Rotterdam University of Applied Sciences, Rotterdam, The Netherlands; 3 Department of Medical Oncology, Erasmus MC Cancer Institute, Rotterdam, The Netherlands; Foundation IRCCS Neurological Institute C. Besta, ITALY

## Abstract

**Purpose:**

Involvement of palliative care experts improves the quality of life and satisfaction with care of patients who are in the last stage of life. However, little is known about the relation between palliative care expert involvement and quality of dying (QOD) in the hospital. We studied the association between palliative care team (PCT) consultation and QOD in the hospital as experienced by relatives.

**Methods:**

We conducted a secondary analysis of data from a prospective study among relatives of patients who died from cancer in a university hospital and compared characteristics and QOD of patients for whom the PCT was or was not consulted.

**Results:**

175 out of 343 (51%) relatives responded to the questionnaire. In multivariable linear regression PCT was associated with a 1.0 point better QOD (95% CI 0.07–1.96). In most of the subdomains of QOD, we found a non-significant trend towards a more favorable outcome for patients for whom the PCT was consulted. Patients for whom the PCT was consulted had more often discussed their preferences for medical treatment, had more often been aware of their imminent death and had more often been at peace with their imminent death. Further, patients for whom the PCT was consulted and their relatives had more often been able to say goodbye. Relatives had also more often been present at the moment of death when a PCT had been consulted.

**Conclusion:**

For patients dying in the hospital, palliative care consultation is associated with a favorable QOD.

## Introduction

Patients with an advanced incurable disease are often admitted to hospital for some time during the last phase of life and a substantial proportion of these patients eventually die in the hospital. Care in hospitals is generally focused at curing disease and prolonging life and may therefore not in all cases adequately address the needs of dying patients. Several studies have reported on shortcomings in the quality of care and unmet needs of patients dying in the hospital, which is e.g. reflected in poor symptom control, the use of aggressive treatments until shortly before dying and a lack of awareness of the approach of death.[1–4]

Involvement of palliative care experts has been shown to be associated with better outcomes for patients with advanced disease.[5] Their involvement was found to improve patients’ quality of life [6–8], their satisfaction with care [8, 9] and communication about their goals of care, resulting in less diagnostic testing and less use of inappropriate technology and intensive care.[10] Studies that assess the association between consultation of palliative care expert teams (PCTs) in hospitals and QOD are scarce. In the Erasmus Medical Center, a university hospital in Rotterdam, clinical specialists can ask the multidisciplinary expert team for pain and palliative care to provide them with advice and support in their patient care. The PCT consists of palliative care nurses, a medical oncologist, a neurologist and a team of anesthesiologists and is available 24/7. The PCT focuses on symptom management, psychosocial support and medical decision making.[11] Upon their involvement, the PCT nurse performs an in-depth assessment of physical, psychosocial and spiritual needs and of the home situation. The PCT does not take over medical treatment but visits the patient daily and provides advice to the treating physician during hospitalization. If specialized psychosocial or spiritual care is needed, the PCT advises the treating physician to consult a psychologist, spiritual caregiver or social worker.

In this observational study we aim to assess whether there is an association between consultation of a PCT in a university hospital and (aspects) of QOD.

## Methods

### Study design and setting

Between June 2009 and July 2012, a questionnaire study was performed among relatives of patients who died in the Erasmus Medical Center, a 1300 bed general university hospital in The Netherlands. We performed a secondary analysis of data from this prospective study which assessed the quality of palliative and terminal care in the hospital, the PalTech-H- study. More information on this study can be found elsewhere.[12, 13]

### Population

The study population consisted of all adult patients who died between June 2009 and July 2012 at one of the 18 non-intensive care wards in the hospital after an admission of at least 6 hours. Both expected and unexpected deaths could be included. Healthcare professionals were not involved in the selection of relatives, but had the opportunity to refuse contacting a relative, which occurred only in three cases.

10–13 weeks after a patient had died on a ward, a relative was invited by the primary investigator (FEW) to complete a questionnaire. In case of no response, a reminder was sent four weeks later. Relatives within a family decided who completed the questionnaire. As the PCT is mainly consulted for patients with cancer, we restricted our analysis for this paper to patients with cancer.

### Questionnaire

A 93 item questionnaire was developed by an expert group to investigate QOD as perceived by relatives. The questionnaire included relevant items from validated questionnaires, including the VOICES (Views of Informal Caregivers Evaluation of Services Scale) [14] and the QODD (Quality of Death and Dying scale).[15] Questions concerned patient characteristics, characteristics of the relative (gender, age, relation to the patient, involvement of relative in care for the patient) and patients’ physical and psychological symptoms during the last 3 days and the last 24 hours of life. Furthermore, the questionnaire included questions on physical, psychological, social and existential experiences, life closure, death preparation, circumstances of death and health care. Overall quality of life and QOD were assessed by asking “How would you evaluate the quality of life during the last 3 days of life of your relative?” and “How would you evaluate the quality of dying of your relative?”. These questions could be answered on a 0–10 numerical scale, with 0 indicating “very poor” and 10 “almost perfect”. The single item measure on QOD was used in several other studies and appeared to be associated with more extensive measures of QOD.[16–18]

Preliminary versions of the questionnaire were critically appraised by a representative of the hospital patient council and tested on relevance and face validity among four relatives of recently passed loved ones. In the first 30 cases the questionnaire was piloted and afterwards some small changes in wording were made.

Patient demographics such as date of birth and gender and underlying diagnosis were retrieved from the medical file. The PCT registry was used to identify whether patients received PCT consultation. This registry contains information on characteristics of the patients that were seen by the PCT, including reasons for consultation and patients’ symptoms. In case the PCT had been involved, we extracted information from the PCT registry regarding the date of their involvement and the reasons for consulting.

### Statistical analysis

The primary outcome measure in this study was QOD. Multivariable linear regression was used to assess the effect of PCT consultation QOD while adjusting for possible confounders.

Second, we assessed the effect of PCT consultation on quality of life in the last three days of life using multivariable linear regression. In order to account for possible correlation between the two main outcome variables, we performed an additional MANCOVA analysis. Third, we compared experiences of relatives of patients who died after PCT consultation and patients who died without such a consultation in a number of subdomains of QOD. We used chi-square tests to assess the statistical significance of differences between the groups. To adjust for multiple testing, we calculated adjusted p-values using the Holm-Bonferroni method.

### Ethical considerations

The Medical Ethical Research Committee of the Erasmus MC approved the study. Participants were given the opportunity to contact the nurse investigator (FEW) in case of emotional distress.

## Results

We received questionnaires from relatives of 175 deceased patients, out of a total of 343 patients with cancer who had died in the hospital during the study period (response 51%). PCT consultation had been provided for 77 out of these 175 patients. Relatives who filled in the questionnaire were mainly women who were the spouse or child of the deceased patient.

### Characteristics of deceased patients and their relatives

Patients for whom the PCT was consulted were younger (p = 0.03) and they more often died in a surgical ward (p<0.01), as compared to patients for whom the PCT was not consulted. (Table 1) Patients for whom the PCT was consulted had more often been ill for over 6 months, but this difference was not statistically significant. We found no significant differences in the duration of latest hospital admission or in the degree of involvement of relatives in informal care during the last 24 hours.

**Table 1:** Characteristics of deceased patients and their relatives (n = 175).

		Without PCT consultation n (%)	With PCT consultation N (%)	P-value±
		N = 98	N = 77	
Patients				
Gender‡	Male	57 (58	52 (68)	0.20
	Female	41 (42)	25 (32)	
Age† (years)	Mean (sd)	69 (12.5)	65 (11.1)	0.03
Marital Status‡	Married/ living with partner	62 (66)	53 (74)	0.29
	Widowed /divorced / living alone/other	32 (34)	19 (26)	
	Missing	0	5	
Education‡	Low	30 (33)	19 (27)	0.30
	Intermediate	43 (48)	35 (49)	
	High	13 (14)	15 (21)	
	Unknown	4 (4)	2 (3)	
	Missing	8	6	
Religious‡	Yes	45 (49)	30 (42)	0.37
Duration of severe illness according to relative‡	≤ 6 Months	43 (46)	25 (34)	0.13
	>6 Months	51 (54)	48 (66)	
	Missing	5	4	
Ward‡	Nonsurgical	78 (80)	46 (60)	<0.01
	Surgical	20 (20)	31 (40)	
Relative involved in in-formal care last 24 hrs‡	Yes	81 (85)	63 (84)	0.85
	No	14 (15)	10 (16)	
	Missing	3	4	
Duration of last admission† (days)	Mean (sd)	13,5 (12,1)	13,7 (16,9)	0.93
Relatives				
Age† (years)	Mean (sd)	57,1 (12,7)	56,1 (12,8)	0.62
Gender‡	Male	37 (39)	25 (35)	0.58
	Female	58 (61)	47 (65)	
	Missing	3	3	
Relation‡	Partner/spouse	44 (48)	42 (60)	0.09
	Child (in law)	38 (41)	18 (26)	
	Other	10 (11)	10 (14)	
	Missing	6	7	

† T-test

‡Chi-square test

± Variables with a difference <0.10 were included in the multivariable model

### Characteristics of PCT consultation

The main symptom for which the PCT was consulted was pain. (Table 2) Pain was among the reasons to involve the PCT in 83% of all cases; other relatively common reasons were constipation or ileus (22%) and dyspnea (19%). Less frequent reasons for consulting the PCT were confusion (6%) and nausea or vomiting (5%).

**Table 2:** Reasons for consulting the palliative care team (n = 77).

	Reasons for consultation† n (%)	Main reason n (%)
Pain	64 (83)	55 (71)
Dyspnea	15 (19)	8 (10)
Confusion / delirium	5 (6)	1 (1)
Constipation / ileus	17 (22)	0 (0)
Nausea / vomiting	4 (5)	0 (0)
Other symptoms	12 (16)	2 (3)
Advice /starting palliative sedation	10 (13)	6 (8)
Advance care planning	8 (10)	5 (7)

†A maximum of 3 reasons for consultation was registered per consultation.

In 10 cases the PCT consultation had occurred during a previous hospital admission, which took place between 16 to 296 days before the admission that ended with the patient’s death. For cases in which the PCT was involved during the final admission, we assessed the time between admission and the first contact with the PCT and the time between the first contact and death. Among these cases, the PCT was consulted on the day of admission in 21% and later in the first week after admission in 55%. In 13% of all cases, the PCT was consulted more than a month before the patient’s death, in 76% within the last two weeks before the patient’s death, and in 9% on the day of death. (Table 3)

**Table 3:** Time between admission and consultation and time between consultation and death† (n = 67).

	Time between moment of admission and first contact with the PCT	Time between first contact with the PCT and death
	n (%)	n (%)
<1 day	14 (21)	6 (9)
1–3 days	23 (34)	18 (27)
4–7 days	14 (21)	14 (21)
8–14 days	7 (10)	13 (19)
15–30 days	4 (6)	7 (10)
31–90 days	5 (7)	7 (10)
>90 days	0 (0)	2 (3)

† For 10 patients, (the latest) PCT consultation had been provided during an admission that preceded the admission that ended with the patient’s death; these patients are not included in the table.

### Patients’ symptoms, quality of life and quality of dying

Relatives were asked to rate the patient’s symptoms during the last three days and the last 24 hours before death. Patients’ symptom burden appeared to be high. The prevalence of moderate or severe pain during the last three days of life was 74% for patients for whom the PCT was consulted compared to 62% for patients for whom the PCT was not consulted; during the last 24 hours of life these prevalences were 65% and 51%, respectively. The prevalence of moderate or severe fatigue during the last three days of life was 85% in both groups, and 85% versus 79% during the last 24 hours of life. The differences in symptom prevalence between patients for whom the PCT was and was not consulted, were not statistically significant. (Table 4)

**Table 4:** Symptoms, quality of life and quality of death according to relatives (n = 175).

	Without PCT consultation N = 98 n (%)	With PCT consultation N = 77 n (%)	P-value
Moderate or severe symptoms in the last 3 days before death:			
Pain	45 (62)	50 (74)	0.11‡
Fatigue	68 (85)	52 (85)	0.97‡
Dyspnea	46 (59)	36 (60)	0.90‡
Anxiety	28 (44)	22 (42)	0.87‡
Agitation	38 (50)	29 (44)	0.47‡
Moderate or severe symptoms in the last 24 hours before death:			
Pain	36 (51)	37 (65)	0.11‡
Fatigue	56 (79)	43(85)	0.92‡
Dyspnea	48 (63)	36 (63)	1.00‡
Anxiety	29 (50)	27 (64)	0.16‡
Agitation	32 (46)	33 (57)	0.24‡
Quality of life and quality of death according to relatives			
Quality of life (mean (sd))	3.72 (2.57)	3.26 (2.76)	0.28†
Quality of dying (mean (sd))	5.82 (2.73)	6.68 (2.64)	0.05†

† T-test

‡ Chi-square

There was no significant difference in relatives’ ratings of patients’ quality of life during the last three days of life. However, their average QOD score for patients with PCT consultation was 6.7 compared to 5.8 for patients for whom the PCT was not consulted (p = 0.05) (Table 4). The multivariable regression model showed that patients for whom the PCT was consulted scored on average one point higher for QOD (95% CI = 0.07–1.96) compared to patients for whom no PCT was consulted (Table 5). There was no significant association between PCT involvement and quality of life. (Table 5) Based on the Wilk’s lambda criterion, the combined dependent variables (QOD and quality of life) were significantly affected by the PCT consultation F (2,140) = 3.89, p = 0.023. Subsequent testing showed a significant effect of PCT on QOD (F (1,141) = 4.54, p = 0.035) but not on quality of life (F (1,141) = 0.77, p = 0.381).

**Table 5:** Multivariable linear regression analysis assessing the effect of patient and treatment characteristics (including PCT consultation) on Quality of Dying and Quality of Life (n = 150).

		Quality of Dying				Quality of life			
		B†	p-value	95% CI		B†	p-value	95% CI	
PCT involvement	PCT	1,00	0,04	0,07	1,96	-0,52	0,25	-1,40	0,36
	No PCT	0				0			
Patients age		0,05	0,05	0,00	0,09	0,02	0,38	-0,02	0,06
Gender	Male	0,30	0,52	-0,62	1,21	-0,09	0,83	-0,98	0,79
	Female	0				0			
Duration of illness	< 6 months	0,30	0,52	-0,61	1,20	1,09	0,01	0,24	1,95
	>6 months	0				0			
Ward	Surgical ward	-0,03	0,96	-1,04	0,99	0,22	0,65	-0,74	1,18
	Non-surgical ward	0				0			
Relatives relation	Partner	0,57	0,42	-0,83	1,97	-0,62	0,35	-1,91	0,68
	Child	0,09	0,91	-1,44	1,63	-1,64	0,03	-3,08	-0,19
	Parent	3,07	0,09	-0,46	6,60	-0,30	0,85	-3,43	2,84
	Other	0				0			

†Data represent change in QOD or quality of life, measured on a scale from 0–10.

### End of life discussions, awareness and life closure

According to relatives, patients for whom the PCT was consulted scored better on several subdomains of QOD: Patients for whom the PCT was consulted had discussed their preferences for medical treatment at the end of life more often than patients for whom the PCT was not consulted, they had more often been aware of the imminence of their death, they had more often been able to say goodbye and they had more often had been at peace with their imminent death. Relatives of patients for whom the PCT was consulted had more often been aware of the imminence of the patient’s death, had more often been able to say goodbye, and had more often been present at the moment of death. However, after the Holm-Bonferroni correction, these differences were not statistically significant, except for the discussion of preferences for medical care with the general practitioner. (Table 6)

**Table 6:** End of life discussions, awareness and life closure according to relatives (n = 175).

		Without PCT consultation n (%)	With PCT consultation n (%)	X^2^	P value†
Patient had discussed preferences for medical treatment at end of life with somebody.	Yes	57 (62)	59 (82)	7.79	<0.01
	No	35 (38)	13 (18)		
	Missing	6	5		
Patient had discussed preferences for medical treatment at end of life with family	Yes	58 (59)	60 (78)	6.89	0.009
	No	40 (41)	17 (22)		
	Missing	0	0		
Patient had discussed preferences for medical care at end of life with a GP	Yes	15 (16)	27 (38)	9.52	0.002
	No	77 (84)	45 (62)		
	Missing	6	5		
Patient had discussed preferences for medical care at end of life with a medical specialist	Yes	24 (26)	27 (38)	2.46	0.117
	No	68 (74)	45 (62)		
	Missing	6	5		
Patient had discussed preferences for medical care at end of life with a nurse	Yes	6 (7)	9 (13)	1.74	0.188
	No	86 (93)	63 (87)		
	Missing	6	5		
Preferences were met?	Yes	12 (48)	13 (52)	0.108	0.743
	No	45 (52)	42 (48)		
	Missing	41	22		
Would the relatives preferred to have more discussions on preferences and medical treatment?	Yes	23 (26)	23 (32)	1.02	0.600
	No	48 (53)	33 (46)		
	DK*	19 (21)	15 (21)		
	Missing	8	6		
Patient was aware of imminent death	Yes	20 (22)	28 (39)	7.02	0.027
	No	60 (64)	32 (45)		
	DK	13 (14)	11 (16)		
	Missing	3	4		
At what moment was the patient aware of imminent death?	>72h	7 (13)	20 (35)	7.95	0.019
	<72h	32 (59)	28 (49)		
	DK	15 (28)	9 (16)		
	Missing	44	20		
Patient was able to say goodbye	Yes	38 (40)	39 (56)	8.03	0.018
	No	55 (59)	27(39)		
	DK	1 (1)	4 (6)		
	Missing	4	7		
Patient was at peace with imminent death	Yes	34 (38)	42 (57)	6.81	0.033
	No	28 (31)	18 (25)		
	DK	28 (31)	13 (18)		
	Missing	8	4		
Relative was aware of imminent death	Yes	37 (40)	43 (59)	6.01	0.048
	No	53 (58)	28 (38)		
	DK	2 (2)	2 (3)		
	Missing	6	4		
At what moment was the relative aware of imminent death?	>72h	20 (32)	30 (48)	3.35	0.067
	<72h	42 (68)	32 (52)		
	Missing	36	15		
Relative said goodbye to patient	Yes	44 (46)	44 (62)	4.00	0.046
	No	51 (54)	27 (38)		
	Missing	3	6		
Relative was present at moment of death	Yes	71 (75)	63 (88)	4.21	0.040
	No	24 (25)	9 (12)		
	Missing	3	5		

† P-values were calculated using the Holm-Bonferroni method

*DK = don’t know

### Hospital care during the last days of life

Several aspects of hospital care were investigated, such as efforts to alleviate symptoms, social support and patients’ and relatives’ participation in medical decision making. We did not find statistically significant differences between patients for whom the PCT was or was not consulted. (Table 7)

**Table 7:** Hospital care in the last days of life according to relatives (n = 175).

		Without PCT consultation n (%)	With PCT consultation n(%)	X^2^	P value†
Efforts to alleviate symptoms and problems last 3 days before death were sufficient	Yes	51 (56)	43 (61)	3.89	0.422
	No	7 (8)	9 (13)		
	Partly	20 (22)	8 (11)		
	NA*	10 (11)	8 (11)		
	DK**	3 (3)	3 (4)		
	Missing	7	6		
Efforts to alleviate symptoms and problems last 24 hours before death were sufficient	Yes	62 (77)	48 (71)	0.53	0.913
	No	9 (10)	7 (10)		
	Partly	13 (15)	10 (15)		
	DK	2 (2)	3 (4)		
	Missing	12	9		
	Missing	21			
Social support the last 3 days before death was sufficient	Yes	49 (54)	32 (46)	4.28	0.370
	No	11 (12)	15 (21)		
	Partly	12 (13)	13 (19)		
	NA	11 (12)	7 (10)		
	DK	7 (8)	3 (4)		
	Missing	8	7		
Social support the last 24 hours before death was sufficient	Yes	54 (61)	43 (66)	3.66	0.301
	No	10 (11)	10 (15)		
	Partly	17 (19)	11 (17)		
	DK	7 (8)	1 (2)		
	Missing	10	12		
In the last days of life, **patient** participated sufficiently in decision making on medical treatment	Yes	45 (52)	34 (50)	0.14	0.987
	No	14 (16)	10 (15)		
	Sometimes	15 (17)	13 (19)		
	DK	14 (16)	11 (16)		
	Missing	10	9		
In the last days of life, **relative** participated sufficiently in decision making on medical treatment	Yes	65 (74)	47(67)	0.97	0.614
	No	17 (19)	18 (26)		
	DK	6 (7)	5 (7)		
	Missing	10	7		
Did the relative receive sufficient information in the last days before death?	Yes	66 (73)	51 (72)	1.60	0.449
	Too much	1(1)	3 (4)		
	Too little	23 (26)	17 (24)		
	Missing	8	6		
Information that was given to the relative was understandable	Yes	71 (79)	49 (68)	2.71	0.439
	No	1 (1)	1 (1)		
	Partly	12 (13)	13 (18)		
	No info	6 (7)	9(13)		
	Missing	8	5		
Relatives were informed about imminent death	Yes	53 (58)	46 (64)	0.54	0.463
	No	38 (42)	26 (36)		
	Missing	7	5		
Opportunity to discuss personal or religious preferences was sufficient	Yes	46 (53)	45 (64)	6.536	0.038
	No	15 (17)	16 (23)		
	DK	26 (30)	9 (13)		
	Missing	11	7		
Attention was paid to personal or religious preferences	Yes	47 (51)	40 (56)	2.60	0.272
	No	7 (8)	10 (14)		
	DK	35 (39)	21 (29)		
	Missing	9	6		
Attention to preferred rituals at the moment of death was sufficient	Yes	40 (49)	36 (58)	3.67	0.159
	No	8 (10)	10 (16)		
	DK	34 (41)	17 (27)		
	Missing				
	Missing	30		
Affirmation of the patient as a whole person was sufficient	Yes	56 (61)	40 (58)	2.02	0.568
	No	8 (9)	6 (9)		
	Partly	19 (12)	12 (17)		
	DK	8 (9)	11 (16)		
	Missing	7	8		
Attention to wishes of patient and relatives in the days before death was sufficient	Yes	63 (70)	55 (77)	2.30	0.513
	No	7 (8)	6 (9)		
	Partly	11 (12)	7 (10)		
	DK	9 (10)	3 (4)		
	Missing	8	6		

† P-values were calculated using the Holm-Bonferroni method

*NA = Not applicable

** Don’t know

64% of relatives of patients for whom the PCT was consulted stated that there had been sufficient opportunity to discuss religious preferences, compared to 53% of relatives of patients without consultation. No significant differences were found regarding the provision of information, attention for preferred rituals at the moment of dying or affirmation of the patient as a whole person.

## Discussion

In this observational study we found an association between involvement of a hospital-based PCT and QOD in patients with cancer. Patients for whom the PCT was or was not consulted were comparable regarding gender, marital status, education, duration of the illness and duration of the latest hospital admission. Patients for whom the PCT was consulted were younger and more often admitted to a surgical ward than patients for whom the PCT was not consulted. In a nationwide Dutch study, it was also found that patients for whom a PCT is consulted are often younger compared to patients for whom the PCT is not consulted.[19] Involvement of the PCT mostly occurred rather late in the disease trajectory: in 76% of all cases the first contact with the PCT occurred within two weeks before death. From other studies it is known that late referral to a PCT is common [20–22], although late referral may decrease the effect of PCT involvement.[23]

The mean QOD score according to relatives for patients for whom the PCT was consulted was 6.7 compared to 5.8 for patients without PCT consultation. This difference remained significant when taking into account potential confounders in a multivariable regression model. This is comparable to an Italian study in which the effect of the Liverpool Care Pathway (LCP) on quality of care for patients with cancer who are dying in the hospital was studied. This study reported a mean score of quality of care at the end of life of 70,5 on a 0–100 scale for patients who died at a ward where the LCP was implemented, compared to a score of 63 for patients on the control wards.[24]

QOD is a multidimensional construct that has been suggested to include physical, psychological, social and spiritual aspects, and issues related to life closure, death preparation and circumstances of death and characteristics of health care at the end of life.[25] We found a non-significant trend towards a more favorable outcome for patients for whom the PCT was consulted such as more discussion of preferences for medical treatment at the end of life, more and earlier awareness of impending death (both in patients and relatives) and more patients being at peace with their imminent death. Relatives were more often able to say goodbye to the patient and more often present at the moment of death. However, these associations were not statistically significant. In other studies, it was found that no or late specialized palliative care involvement is associated with worse death preparation [26] and decreased disease awareness of terminally ill patients.[27]

We did not find a statistically significant difference in quality of the last three days of life. In several other studies, positive effects of PCT involvement on patients’ quality of life were found. In these studies, contrary to our study, the PCT was involved relatively early in patients’ disease trajectory and quality of life was not assessed during the last days before death.[6, 28, 29]

The PCT was mainly consulted for physical symptoms; the most frequently mentioned reason for involving the PCT was pain, followed by dyspnea, which is also in line with other studies.[30–32] The PCT that was studied always performs a multidimensional assessment of the patient’s condition and needs, even if the initial reason for consulting the PCT is related to pain problems. The PCT assesses physical, social, psychological and spiritual problems and discusses these with the treating physician. We found no significant differences in the severity of patients’ symptoms during the days before death. As we did not conduct before and after measurements of symptoms, we cannot draw any conclusions on the impact of involvement of the PCT on symptom burden. Nevertheless, symptom burden in patients for whom the PCT was consulted may have been higher at admission compared to patients for whom the PCT was not consulted, as pain was often the reason for consulting the PCT. Furthermore, in 38% of consultations, the PCT was consulted within the last 3 days of life, which may represent a timeframe that is too short to have a significant impact on symptoms. Finally, involvement of the PCT can also be related to a specialist’s awareness of the availability of the PCT or their willingness to consult the PCT.[33]

### Limitations

The explorative nature of this study implies that we cannot draw strong conclusions about the causal relation between the involvement of PCT and aspects of QOD. There may be other factors besides the involvement of the PCT that account for the differences in QOD that were found in this study, such as prior awareness and communication and confounding by indication. Second, as we performed a secondary analysis of existing data, the power of the study may have been insufficient to detect statistically significant differences between both groups. Furthermore, this study is restricted to the perspectives of the relatives. From other research it is known that perspectives of relatives can differ from those of the patient or the physician.[34] We did not have information on the non-responders, so selection bias cannot be ruled out. As this study was performed in a single, academic centre, the generalizability of the findings may be limited.

## Conclusion

In this study, we found that PCT consultation was associated with a favorable QOD for patients with cancer who died in the hospital. Our results suggest that PCT involvement has positive effects on patients’ and relatives’ awareness of death.

## Supporting information

S1 Questionnaire Questionnaire Relatives _MaleVersionEnglish.doc. (DOC)
