# Correction: Palliative care team consultation and quality of death and dying in a university hospital: A secondary analysis of a prospective study

**DOI:** 10.1371/journal.pone.0208564

**Published:** 2018-11-29

**Authors:** Arianne Brinkman-Stoppelenburg, Frederika E. Witkamp, Lia van Zuylen, Carin C. D. van der Rijt, Agnes van der Heide

In Tables 6 and 7, the p-values presented did not reflect the Holm Bonferroni correction. The text and conclusions of the article are based on the corrected p-values. Please see the corrected Tables 6 and 7 here.

**Table 6:** End of life discussions, awareness and life closure according to relatives (N = 175).

		Without PCT consultation n (%)	With PCT consultation n(%)	X^2^	P value†
Patient had discussed preferences for medical treatment at end of life with somebody.	Yes	57 (62)	59 (82)	7.79	0.070
	No	35 (38)	13 (18)		
	Missing	6	5		
Patient had discussed preferences for medical treatment at end of life with family	Yes	58 (59)	60 (78)	6.89	0.117
	No	40 (41)	17 (22)		
	Missing	0	0		
Patient had discussed preferences for medical care at end of life with a GP	Yes	15 (16)	27 (38)	9.52	0.030
	No	77 (84)	45 (62)		
	Missing	6	5		
Patient had discussed preferences for medical care at end of life with a medical specialist	Yes	24 (26)	27 (38)	2.46	0.468
	No	68 (74)	45 (62)		
	Missing	6	5		
Patient had discussed preferences for medical care at end of life with a nurse	Yes	6 (7)	9 (13)	1.74	0.564
	No	86 (93)	63 (87)		
	Missing	6	5		
Preferences were met?	Yes	12 (48)	13 (52)	0.108	1.000
	No	45 (52)	42 (48)		
	Missing	41	22		
Would the relatives preferred to have more discussions on preferences and medical treatment?	Yes	23 (26)	23 (32)	1.02	1.000
	No	48 (53)	33 (46)		
	DK*	19 (21)	15 (21)		
	Missing	8	6		
Patient was aware of imminent death	Yes	20 (22)	28 (39)	7.02	0.270
	No	60 (64)	32 (45)		
	DK	13 (14)	11 (16)		
	Missing	3	4		
At what moment was the patient aware of imminent death?	>72h	7 (13)	20 (35)	7.95	0.216
	<72h	32 (59)	28 (49)		
	DK	15 (28)	9 (16)		
	Missing	44	20		
Patient was able to say goodbye	Yes	38 (40)	39 (56)	8.03	0.216
	No	55 (59)	27(39)		
	DK	1 (1)	4 (6)		
	Missing	4	7		
Patient was at peace with imminent death	Yes	34 (38)	42 (57)	6.81	0.297
	No	28 (31)	18 (25)		
	DK	28 (31)	13 (18)		
	Missing	8	4		
Relative was aware of imminent death	Yes	37 (40)	43 (59)	6.01	0.322
	No	53 (58)	28 (38)		
	DK	2 (2)	2 (3)		
	Missing	6	4		
At what moment was the relative aware of imminent death?	>72h	20 (32)	30 (48)	3.35	0.335
	<72h	42 (68)	32 (52)		
	Missing	36	15		
Relative said goodbye to patient	Yes	44 (46)	44 (62)	4.00	0.322
	No	51 (54)	27 (38)		
	Missing	3	6		
Relative was present at moment of death	Yes	71 (75)	63 (88)	4.21	0.320
	No	24 (25)	9 (12)		
	Missing	3	5		

^†^ P-values were calculated using the Holm-Bonferroni method

*DK = don’t know

**Table 7:** Hospital care in the last days of life according to relatives (N = 175).

		Without PCT consultation n (%)	With PCT consultation n(%)	X^2^	P value†
Efforts to alleviate symptoms and problems last 3 days before death were sufficient	Yes	51 (56)	43 (61)	3.89	1.000
	No	7 (8)	9 (13)		
	Partly	20 (22)	8 (11)		
	NA*	10 (11)	8 (11)		
	DK**	3 (3)	3 (4)		
	Missing	7	6		
Efforts to alleviate symptoms and problems last 24 hours before death were sufficient	Yes	62 (77)	48 (71)	0.53	1.000
	No	9 (10)	7 (10)		
	Partly	13 (15)	10 (15)		
	DK	2 (2)	3 (4)		
	Missing	12	9		
Social support the last 3 days before death were sufficient	Yes	49 (54)	32 (46)	4.28	1.000
	No	11 (12)	15 (21)		
	Partly	12 (13)	13 (19)		
	NA	11 (12)	7 (10)		
	DK	7 (8)	3 (4)		
	Missing	8	7		
Social support the last 24 hours before death were sufficient	Yes	54 (61)	43 (66)	3.66	1.000
	No	10 (11)	10 (15)		
	Partly	17 (19)	11 (17)		
	DK	7 (8)	1 (2)		
	Missing	10	12		
In the last days of life, **patient** participated sufficiently in decision making on medical treatment	Yes	45 (52)	34 (50)	0.14	1.000
	No	14 (16)	10 (15)		
	Sometimes	15 (17)	13 (19)		
	DK	14 (16)	11 (16)		
	Missing	10	9		
In the last days of life, **relative** participated sufficiently in decision making on medical treatment	Yes	65 (74)	47(67)	0.97	1.000
	No	17 (19)	18 (26)		
	DK	6 (7)	5 (7)		
	Missing	10	7		
Did the relative receive sufficient information in the last days before death?	Yes	66 (73)	51 (72)	1.60	1.000
	Too much	1 (1)	3 (4)		
	Too little	23 (26)	17 (24)		
	Missing	8	6		
Information that was given to the relative was understandable	Yes	71 (79)	49 (68)	2.71	1.000
	No	1 (1)	1 (1)		
	Partly	12 (13)	13 (18)		
	No info	6 (7)	9 (13)		
	Missing	8	5		
Relatives were informed about imminent death	Yes	53 (58)	46 (64)	0.54	1.000
	No	38 (42)	26 (36)		
	Missing	7	5		
Opportunity to discuss personal or religious preferences was sufficient	Yes	46 (53)	45 (64)	6.536	0.532
	No	15 (17)	16 (23)		
	DK	26 (30)	9 (13)		
	Missing	11	7		
Attention was paid to personal or religious preferences	Yes	47 (51)	40 (56)	2.60	1.000
	No	7 (8)	10 (14)		
	DK	35 (39)	21 (29)		
	Missing	9	6		
Attention to preferred rituals at the moment of death was sufficient	Yes	40 (49)	36 (58)	3.67	1.000
	No	8 (10)	10 (16)		
	DK	34 (41)	17 (27)		
	Missing	16	14		
Affirmation of the patient as a whole person was sufficient	Yes	56 (61)	40 (58)	2.02	1.000
	No	8 (9)	6 (9)		
	Partly	19 (12)	12 (17)		
	DK	8 (9)	11 (16)		
	Missing	7	8		
Attention to wishes of patient and relatives in the days before death was sufficient	Yes	63 (70)	55 (77)	2.30	1.000
	No	7 (8)	6 (9)		
	Partly	11 (12)	7 (10)		
	DK	9 (10)	3 (4)		
	Missing	8	6		

^†^ P-values were calculated using the Holm-Bonferroni method

*NA = Not applicable

** Don’t know
