# Supplementary material for: Palliative care team consultation and quality of death and dying in a university hospital: A secondary analysis of a prospective study
Source: PLoS One. 2018 Aug 23;13(8):e0201191. doi: 10.1371/journal.pone.0201191 (PMC6107115; doi:10.1371/journal.pone.0201191)
Supplement: S1 Questionnaire — (DOC) [file pone.0201191.s001.doc]

Department of Public Health
 Department of Medical Oncology

# Quality of dying in the hospital

## A scientific study on the quality of care

## in the dying phase at Erasmus MC

Questionnaire for relatives of deceased patients

# *Note: translation into English version only for publication.*

Respondent number:

Erasmus MC zorgonderzoek ‘Kwaliteit van zorg in de stervensfase‘


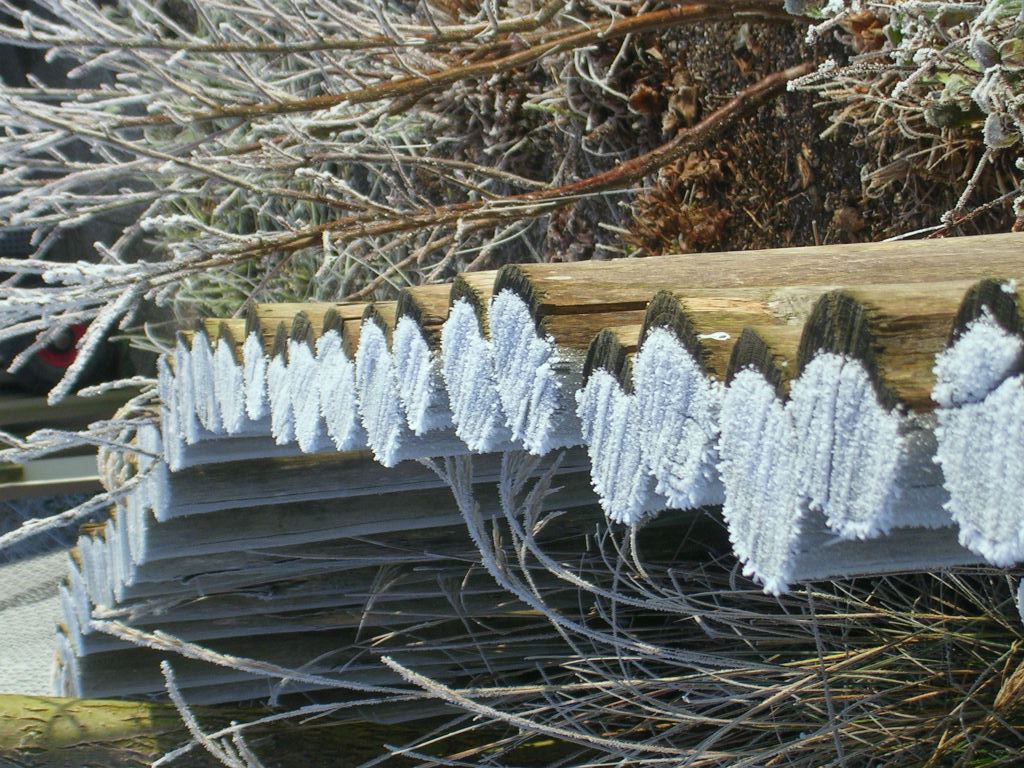


Erasmus MC research: “Quality of care for the dying’

This research is carried out by the Department of Public Health and the Department of Medical Oncology of Erasmus MC.

**Research group:**

Mrs. F.E. Witkamp, nurse researcher

Mrs. dr. A. van der Heide, phsycian / epidemiologist

Mrs. dr. L. van Zuylen, medical oncologist

Prof. dr P.J. van der Maas, professor of Public Health

**Introduction:**

In this survey, the experiences of yourself and of your relative during his last days of life are central. With the information you provide, we can study how you and your relative experienced the last days of his life and the dying phase. We can also study if good care was provided and whether there are points of reference for improving this care.

This questionnaire contains different types of questions:

• in most questions you can choose from a number of answers. For each question, choose the answer you find the most applicable and tick the corresponding box. Sometimes you can tick more than one answer. In that case this is mentioned.

• in addition, there are some questions where you can fill in an answer. You can use the space within the specified lines and frames.

In the questionnaire is regularly talked about 'caregivers’. Here, everyone who provides professional help or care is meant. This includes doctors and nurses, but also the food assistant, physical therapist, dietitian, psychologist, priest or pastor, social worker, etc.

In the questionnaire, questions are asked about experiences in the last days before death. Sometimes it is about "the last days". You can then think of about the last half week before your relative died. At other questions we would like to know how it was on the very last day (last 24 hours) and how it was 3 days before his death (on day 3, so 72 – 48 hours before his death). Several questions concern the experiences at Erasmus MC. In that case this is explicitly mentioned.

Would you please do not skip any questions. If you are in any doubt, please choose the answer closest to your situation. There are no 'good' or 'bad' answers; it comes to the personal experiences of you and your relative in his last phase of life.

Some questions might be impossible to answer because they do not fit your situation. If you find that you are unable to answer a question, please answer n/a (not applicable) to the question.

Erasmus MC zorgonderzoek ‘Kwaliteit van zorg in de stervensfase‘


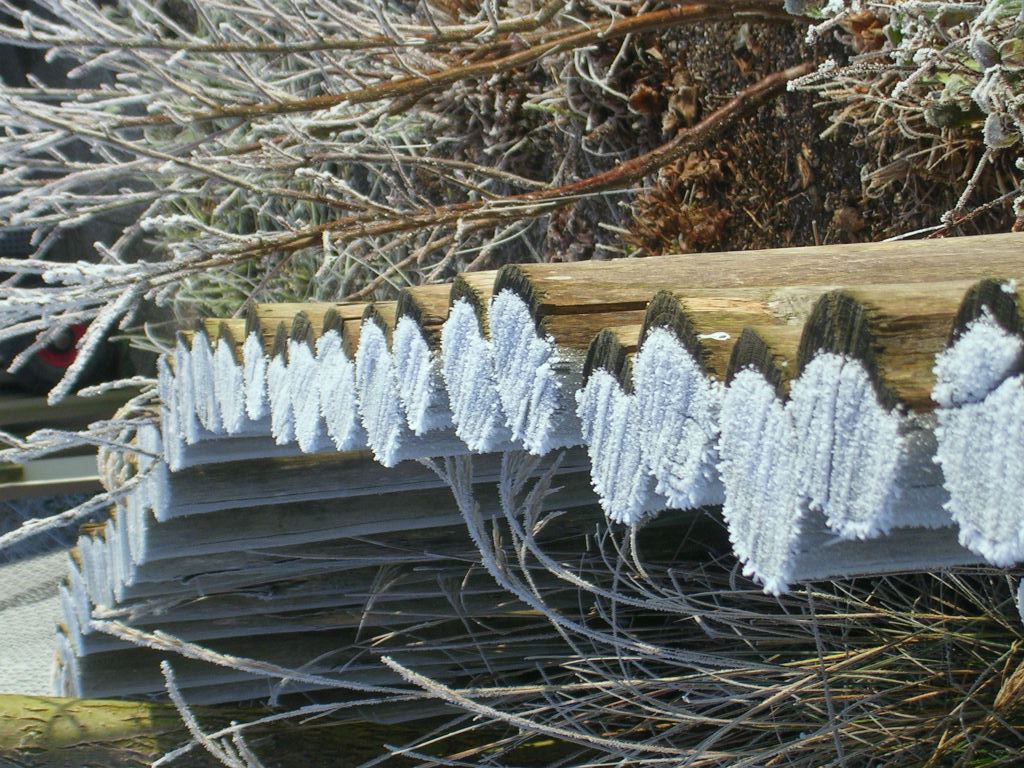


Erasmus MC research: “Quality of care for the dying’

If, after completing this questionnaire, you feel the need to talk about it with the researcher, you can contact her using the details below.

After you returned the questionnaire, your personal information will be anonymized, so that no one can figure out that it came from you.

**If you have any questions or concerns, please contact:**

Mrs. F.E. Witkamp

Erasmus MC, Universitair Medical Center Rotterdam

Department of Public Health

P.O. Box 2040

3000 CA Rotterdam

[f.witkamp@erasmusmc.nl](mailto:L.Veerbeek@erasmusmc.nl)

If you decide not to participate in this research, would you please indicate the reason below?

In this case, would you please answer the first 3 pages of the questionnaire and return it in the attached envelope? We then will send you no reminder.

**We thank you in advance very much for the effort!**

For this research, the Medical Ethics Review Committee of the Erasmus MC issued a declaration of no objection.

Erasmus MC zorgonderzoek ‘Kwaliteit van zorg in de stervensfase‘


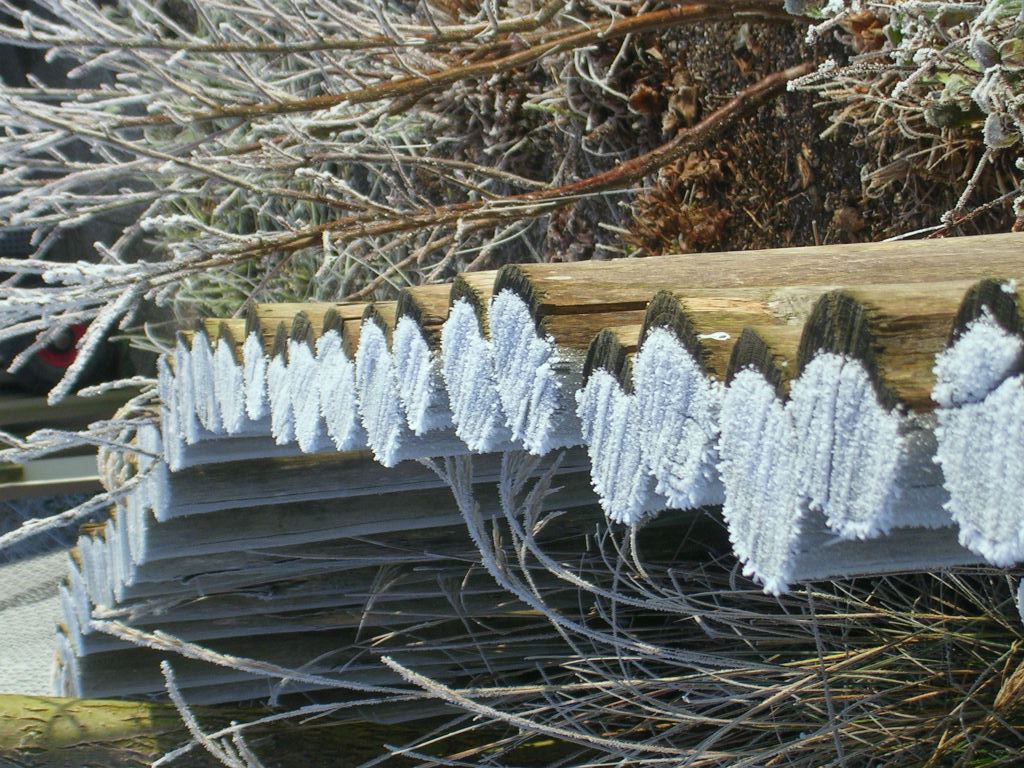


Erasmus MC research: “Quality of care for the dying’

| **A** | **General**  To start, we'll ask you for some general information about your relative and yourself. | | | | | | | | | | **Ref.nr.** | | **W-Code**  **-** |
| --- | --- | --- | --- | --- | --- | --- | --- | --- | --- | --- | --- | --- | --- |
| **A1** | What is the date of birth of your relative?  day month year | | | | | | | | | | | | |
| **A2** | **When did your relative die?**  day month year | | | | | | | | | | | | |
| **A3** | What was his nationality?  Dutch  other, nl.  (you can fill in his nationality here) | | | | | | | | | | | | |
| **A4** | **What was his marital status?**  married or living together  widower  divorced  single  other | | | | | | | | | | | | |
| **A5** | **Did your relative have children?**  yes  no *→ you can continue to question A7* | | | | | | | | | | | | |
| **A6** | **What age do this child or these children have?**  *(you can tick one or more answers)*  0-12 year number of children in this age:  13-18 year number of children in this age:  19 year and older number of children in this age: | | | | | | | | | | | | |
| **A7** | **How was the living arrangement of your relative?** | | | | | | | | | | | | |
|  | independent alone  with partner  with spouse and child/children | | | | without partner with child / children  at parent(s)  else | | | | | | | | |
| **A8** | **What was his highest education?** | | | | | | | | | | | | |
|  | Low (ISCED level 1-2)  Intermediate (ISCED level 3-4) | | | | High (ISCED 5-6)  Other / unknown | | | | | | | | |
| **A9** | **Was your relative religious?**  yes  no *→ you can continue to question A 11*  he had another conviction  I don’t know*→ you can continue to question A 11* | | | | | | | | | | | | |
| **A10** | **What faith / what religion?**  Roman Catholic  Protestant  Islamic  Buddhist  Hindu  Other, nl.:  (you can fill in his faith or belief)  I don’t know | | | | | | | | | | | | |
| **A11** | **What relation did you have with your relative?**  I am his partner / spouse  I am his child  I am his parent  other, nl.:  (you can fill in your relation) | | | | | | | | | | | | |
| **A12** | **What is your age?**  year | | | | | | | | | | | | |
| **A13** | **Are you a male of a female?**  male  female | | | | | | | | | | | | |
| **A14** | **How is your health in general?**  very good  good  average  sometime good, sometimes bad  bad | | | | | | | | | | | | |
| **B** | **De last phase of life**  *The following questions cover the period before the death of your relative* | | | | | | | | | | | | |
| **B1** | **Since when did your relative have serious health problems?**  less than 1 month before his death  more than one month, but shorter than 3 months before his death  between 3 and 6 months before his death  more than half a year, but less than 2 years before his death  more than 2 years before his death | | | | | | | | | | | | |
| **B2** | **What disease (s) or condition (s) these were causing problems?**  You can fill in the disease(s) or condition(s) here**:** | | | | | | | | | | | | |
| **B3** | **Did your relative discuss his preferences for medical treatment at the end of life?**  *(you can tick one or more answers)* | | | | | | | | | | | | |
|  | yes, with: | partner  children  other relatives  friends  general practitioner | | | | specialist  nursing home physician  nurse  someone else | | | | | | | |
|  | no *→ you can continue to question B6* | | | | | | | | | | | | |
| **B4** | **If yes, do you know what preferences were discussed?**  yes, nl.:  (you can fill in his preferences here)  no*→ you can continue to question B6* | | | | | | | | | | | | |
| **B5** | **Were these preferences met?**  yes  no | | | | | | | | | | | | |
| **B6** | **Do you think your relative, in the last month before his death, needed one or more (further) conversations with a physician about his preference regarding medical treatment in the last phase of life?**  yes  no *→ you can continue to question B8*  I don’t know*→ you can continue to questionB8* | | | | | | | | | | | | |
| **B7** | **If yes, do you know about what preferences he would have wanted to discuss?**  yes, nl.:  (you can fill in his preferences here)  no | | | | | | | | | | | | |
| **B8** | **Did your relative finalize affairs related to his illness and impending death?** *Affairs not concerning* *medical decisions, e.g.: to undertake activities which always had been postponed, recover lost contacts, arrange the funeral, saying goodbye, taking care of inheritance, etc.*  yes  no  **Can you please**  **comment?** | | | | | | | | | | | | |
| **B9** | **Has your relative ever stated where he would prefer to die?**  yes  no *→ you can continue to question B11*  I don’t know*→ you can continue to question B11* | | | | | | | | | | | | |
| **B10** | **If yes, where did your relative preferred to die?**  at home  in a nursing home or care home  in a hospice  in a hospital  somewhere else, nl.:  *→ you can continue to question B12* | | | | | | | | | | | | |
| **B11** | **Where did you think your relative had preferred to die?**  at home  in nursing home or in a care home  in a hospice  in a hospital  somewhere else, nl.: | | | | | | | | | | | | |
| **B12** | **In hindsight, do you think the Erasmus MC was the right place to die for your relative?**  yes, it was the right place for him *→ you can continue to question C1*  more or less but (there were no other options)  no, it was not the right place for him  I don’t know*→ you can continue to question C1* | | | | | | | | | | | | |
| **B13** | **It was not quite the right place to die for him because**  he preferred to die somewhere else  he was not well care for there  it was too far away for family and friends  another reason, nl.:  (you can fill in the reason here) | | | | | | | | | | | | |
|  | **The last few days** | | | | | | | | | | | | |
| **C1** | **Were you aware of his imminent death?**  yes  more or less  no *→ you can continue to question C3*  I don’t know*→ you can continue to question C3* | | | | | | | | | | | | |
| **C2** | **At what moment did you became aware of his imminent death?**  more than 3 days before his death  on day 3 (72 - 48 hours) before his death  on day 2 (48 - 24 hours) before his death  less than 24 hours before his death  *→ you can continue to question C4* | | | | | | | | | | | | |
| **C3** | **If it was not clear to you beforehand that your relative would die within a few days, what do you think was the cause?**  *(you can tick one or more answers)*  **he was sick, but I had no reason to think that he would die within a few days**  **suddenly it went very badly with him (due to a complication or an acute situation)**  **I think the doctor did not see this coming either**  **I was not told that it was that bad with him**  **I did not want to believe that it was that bad with him**  Other, nl.:  (you can fill in one or more causes) | | | | | | | | | | | | |
| **C4** | **Was your relative aware of his imminent death?**  yes  more or less  no *→ you can continue to question C7*  I don’t know*→ you can continue to question C7* | | | | | | | | | | | | |
| **C5** | **At what moment was your relative aware of his imminent death?**  more than 3 days before his death  on day 3 (72 - 48 hours) before his death  on day 2 (48 - 24 hours) before his death  less than 24 hours before his death  I don’t know | | | | | | | | | | | | |
| **C6** | **Did you talk to your relative about his approaching death?**  yes *→ you can continue to question C8*  no | | | | | | | | | | | | |
| **C7** | **Would you like to have talked to your relative over about his imminent death?**  yes  no  I don’t know | | | | | | | | | | | | |
| **C8** | **Did you still spoke with your relative shortly before his death or otherwise had contact?**  *(thick on each line the box that applies)* | | | | | | | | | | | | |
|  |  | | **I talked to my relative** | **I did not spoke with my relative, but I did have contact** | | | | | | **I did see my relative, but I had no contact** | | | **I did not see my relative** |
|  | On day 3 (72 – 48 hours) before his death | |  |  | | | | | |  | | |  |
|  | On day 2 (48 – 24 hours) before his death | |  |  | | | | | |  | | |  |
|  | 24 – 12 hours before his death | |  |  | | | | | |  | | |  |
|  | The last 12 hours before his death | |  |  | | | | | |  | | |  |
| **C9** | **Do you think there was something you could do for your relative during the last 24 hours?**  yes  no  I don’t know  **Can you please comment?** | | | | | | | | | | | | |
| **C10** | **Were you able to say good-bye?**  yes  no  more or less | | | | | | | | | | | | |
| **C11** | **Was your relative able to say good-bye to his loved ones?**  yes  no  more or less  I don’t know | | | | | | | | | | | | |
| **C12** | **Were you present at the moment of death of your relative?**  yes *→ you can continue to question C14*  no, but another relative was present  no, there was no relative present | | | | | | | | | | | | |
| **C13** | **If not, would you like to have been present at the moment of death of your relative?**  yes  no  other  **Can you please comment?** | | | | | | | | | | | | |
| **C14** | **Which people from the immediate environment were involved in caring for your relative?**  *(you can tick one or more answers)* | | | | | | | | | | | | |
|  | **A. On day 3 before his death**  *(so ± 72 - 48 hours before death)*  yourself  partner  child(ren)  friends / acquaintance  other relatives  neighbours  volunteers  no one  other person | | | | **B. During the last 24 hours**  yourself  partner  child(ren)  friends / acquaintance  other relatives  neighbours  volunteers  no one  other person | | | | | | | | |
| **D** | **Problems, complaints and symptoms during the last days**  *In the next questions we will address possible problems, complaints of your relative in the last few days.* | | | | | | | | | | | | |
| **D1** | **Was your relative conscious before death?**  *(thick on each line the box that applies)* | | | | | | | | | | | | |
|  |  | | | | | | **yes** | | **partly** | | | **no** | |
|  | on day 3 (72 – 48 hours) before his death | | | | | |  | |  | | |  | |
|  | on day 2 (48 – 24 hours) before his death | | | | | |  | |  | | |  | |
|  | 24 – 12 hours before his death | | | | | |  | |  | | |  | |
|  | the last 12 hours before his death | | | | | |  | |  | | |  | |
|  |  |  |  |  |  |  |  |  |  |  |  |  |  |
| **D2** | **Did you relative have one or more of the below mentioned problems, complaints or symptoms at the third day before his death (so ± 72 -48 hours before his death)? (thick on each line of the box that applies)** | | | | | | | | | | | | |
|  |  | | **None** | **Mild** | | | | **Moderate** | | | **Severe** | | **I don’t know** |
|  | **Pain** | |  |  | | | |  | | |  | |  |
|  | **Dyspnea** | |  |  | | | |  | | |  | |  |
|  | **Coughing** | |  |  | | | |  | | |  | |  |
|  | **Death rattle** | |  |  | | | |  | | |  | |  |
|  | **Difficulty sleeping** | |  |  | | | |  | | |  | |  |
|  | **Fatigue** | |  |  | | | |  | | |  | |  |
|  | **Dry mouth** | |  |  | | | |  | | |  | |  |
|  | **Lack of appetite** | |  |  | | | |  | | |  | |  |
|  | **Nausea** | |  |  | | | |  | | |  | |  |
|  | **Swallowing problems** | |  |  | | | |  | | |  | |  |
|  | **Constipation** | |  |  | | | |  | | |  | |  |
|  | **Decreased consciousness** | |  |  | | | |  | | |  | |  |
|  | **Confusion** | |  |  | | | |  | | |  | |  |
|  | **Agitation** | |  |  | | | |  | | |  | |  |
|  | **Anxiety** | |  |  | | | |  | | |  | |  |
|  | **Loneliness** | |  |  | | | |  | | |  | |  |
|  | **Dependency** | |  |  | | | |  | | |  | |  |
|  | **Tenseness** | |  |  | | | |  | | |  | |  |
|  | **Worrying** | |  |  | | | |  | | |  | |  |
|  | **Sadness** | |  |  | | | |  | | |  | |  |
|  | **Feelings of powerlessness** | |  |  | | | |  | | |  | |  |
|  | **Depressed mood** | |  |  | | | |  | | |  | |  |
| **D3** | **Did you think that the signs or symptoms were sufficiently under control on day 3 before his death?**  yes, he was not much bothered by them  yes, symptoms and problems were sufficiently treated  yes, he was held unconscious  no, he suffered from (one or more) symptoms or problems  I don’t know  other  **Can you please comment?** | | | | | | | | | | | | |

| **D4** | **Did you relative have one or more of the below mentioned problems, complaints or symptoms during the last day before his death (so ± the last 24 hours of his life)? (thick on each line of the box that applies)** | | | | | |
| --- | --- | --- | --- | --- | --- | --- |
|  |  | **None** | **Mild** | **Moderate** | **Severe** | **I don’t know** |
|  | **Pain** |  |  |  |  |  |
|  | **Dyspnea** |  |  |  |  |  |
|  | **Coughing** |  |  |  |  |  |
|  | **Death rattle** |  |  |  |  |  |
|  | **Difficulty sleeping** |  |  |  |  |  |
|  | **Fatigue** |  |  |  |  |  |
|  | **Dry mouth** |  |  |  |  |  |
|  | **Lack of appetite** |  |  |  |  |  |
|  | **Nausea** |  |  |  |  |  |
|  | **Swallowing problems** |  |  |  |  |  |
|  | **Constipation** |  |  |  |  |  |
|  | **Decreased consciousness** |  |  |  |  |  |
|  | **Confusion** |  |  |  |  |  |
|  | **Agitation** |  |  |  |  |  |
|  | **Anxiety** |  |  |  |  |  |
|  | **Loneliness** |  |  |  |  |  |
|  | **Dependency** |  |  |  |  |  |
|  | **Tenseness** |  |  |  |  |  |
|  | **Worrying** |  |  |  |  |  |
|  | **Sadness** |  |  |  |  |  |
|  | **Feelings of powerlessness** |  |  |  |  |  |
|  | **Depressed mood** |  |  |  |  |  |
| **D5** | **Did you think that the signs or symptoms the last day (the last 24 hours) were sufficiently under control?**  yes, he was not much bothered by them  yes, symptoms and problems were sufficiently treated  yes, he was held unconscious  no, he suffered from (one or more) symptoms or problems  I don’t k**n**ow  other  **Can you please**  **comment?** | | | | | |
| **D6** | **Did your relative have any practical problems during the last 3 days (financial or personal, such as caring for children, for a pet or for a company)?**  yes, these problems were sufficiently solved  yes, these problems were not all solved  no  I don’t know | | | | | |
| **D7** | **Did your relative in the last days need the proximity or advice of the people close to him?**  much need  some need  no need *→ you can continue to question D9*  I don’t know*→ you can continue to question D9* | | | | | |
| **D8** | **Do you think that this need had been met?**  yes, largely  yes, somewhat  no  I don’t know | | | | | |
| **D9** | **Was your relative at peace with imminent death?**  yes  no  I don’t know | | | | | |

| **E** | **Care and treatment during the last day in the university hospital**  **The following questions are about the care and treatment of your relative at the university hospital, short before and around the moment of death. Preliminary remark: At various items stressed to the situation at University hospital is often asked about the situation on: a. day 3 before his death (so about 72 – 48 hours before he died) and b. during the last 24 hours. If your relative was admitted to hospital less than 48 hours before his death, put a cross at "not applicable (n/a): he was not yet admitted to the university hospital “at the relevant question.** | | |
| --- | --- | --- | --- |
| **E1** | **Do you think efforts to alleviate symptoms, complaints and problems were sufficient?** | | |
|  | **A. On day 3 before his death**  *(so ± 72 - 48 hours before he died)*  n/a.: he was not yet admitted to the university hospital  yes  partly  no  I don’t know | **B. During the last 24 hours?**  yes  partly  no  I don’t know | |
|  | **Could you please**  **comment?** | | |
| **E2** | **Do you think that sufficient help was available to your relative at the university hospital for nursing care (washing, eating, medicine use, etc.)** | | |
|  | **A. On day 3 before his death**  *(so ± 72 - 48 hours before he died)*  n/a.; he was not yet admitted to the university hospital  yes  partly  no  I don’t know | | **B. During the last 24 hours**  yes  partly  no  I don’t know |
|  | **Could you please**  **comment?** | | |
| **E3** | **Do you think that sufficient social support has been given to your relative and to you in the university hospital (when dealing with the approaching farewell, questions of life, practical problems etc.)?** | | |
|  | **A. On day 3 before his death**  *(so ± 72 - 48 hours before death)*  n/a.; he was not yet admitted to the University hospital  yes  partly  no  I don’t know | | **B. During the last 24 hours**  yes  partly  no  I don’t know |
|  | **Can you please**  **comment?** | | |
| **E4** | **Were you told at the university hospital that your relative would probably die within a few days?**  yes  no *→ you can continue to question E9* | | |
| **E5** | **When were you told at the university hospital that your relative would probably die within a few days?**  more than 3 days before his death  on day 3 (72 - 48 hours) before his death  on day 2 (48 - 24 hours) before his death  less than 24 hours before his death | | |
| **E6** | **Were you, when this was told to you at the university hospital, given the opportunity to talk about this?**  yes  no  I don’t know | | |
| **E7** | **Do you think that you had enough privacy at the university hospital when it was told to you?**  yes  no  I don’t know | | |
| **E8** | **How much 'support' gave it that you were told you that your relative would die?**  a lot  a little  none  *→ you can continue to question E 10* | | |
| **E9** | **Would you have wanted to know in advance that your relative would probably die within a few days?**  yes  no  I don’t know | | |
| **E10** | **Do you have the idea that medical care changed during the last days before the death of your relative?**  yes  no  I don’t know  **Can you please**  **comment?** | | |
| **E11** | **Do you think that you participated sufficiently in decision making at the university hospital during the last days before his death** | | |
|  | **A. in medical treatment?**  yes  no, I would have liked to be more involved  I don’t know | **B. in nursing care?**  yes  no, I would have liked to be more involved  I don’t know | |
|  | **Can you please**  **comment?** | | |
| **E12** | **Do you think that your relative participated sufficiently in decision making at the university hospital during the last days before his death?** | | |
|  | **A. in medical treatment?**  yes  sometimes  no  I don’t know | **B. in nursing care?**  yes  sometimes  no  I don’t know | |
|  | **Can you please**  **comment?** | | |
| **E13** | **Did you receive sufficient information in the university hospital in the last days before death about the situation and care of your relative?**  too much  sufficient  too little | | |
| **E14** | **Was the information you received well understandable?**  yes  partly  no  I did not receive any information | | |
| **E15** | **Did you have the opportunity in the university hospital to discuss personal or religious matters that were of interest to your relative?**  yes  no  I don’t know | | |

| **E16** | **Do you think the opportunity to discuss personal or religious preferences was sufficient?**  yes  no  I don’t know |
| --- | --- |
| **E17** | **Do you think the attention to preferred rituals at the moment of death in the university hospital was sufficient (e.g. regarding religion or culture)?**  yes  no  I don’t know |
| **E18** | **Do you think that there was enough (real) attention at the university hospital for your relative as a person? (affirmation of who he was, with his own personality, beliefs, way of life, etc.)**  yes  partly  no  I don’t know  **Can you please**  **comment?** |
| **E19** | **Do you think that you had enough opportunity at the university hospital to be present with your relative in the last days before his death?**  yes  no  I don’t know |
| **E20** | **Do you think that the attention to wishes of you and your relative in the last days before his death have been sufficiently taken into account at the university hospital? (eg privacy, rest, (wishing) meals, accommodation)**  yes  partly  no  I don’t know  Can you please  comment? |
| **E21** | **Did you have enough information in the university hospital about the usual course of events in the department shortly before and after the death of a patient?**  yes  no  I don’t know |

| **E22** | **Were you involved in looking after your relative after his death?**  yes *→ you can continue to question E 24*  no | | | |
| --- | --- | --- | --- | --- |
| **E23** | **Would you have preferred to have been involved in looking after your loved one after his death?**  yes, but he was not taken care of in the department  yes, but no opportunity was given me  yes, in retrospect, I did, but at that moment I did not want to  no  other  Can you please  comment? | | | |
| **E24** | **Did you, after the death of your relative, talk to a healthcare provider at the university hospital about your feelings regarding his illness / condition and death?**  *(you can tick one or more answers)*  yes, immediately after his death  yes, after a few weeks  no *→ you can continue to question E 26* | | | |
| **E25** | **If yes, / were these conversations useful for you?**  no  I don’t know  **Can you please**  **comment?**  *→ you can continue to question E 27* | | | |
| **E26** | **If no, would you have appreciated talking to someone?**  yes  no  I don’t know | | | |
| **E27** | **Was there anything the caregivers in the university hospital could have done to make the last 3 days of life of your relative more bearable?** | | | |
|  | **A. for your relative?**  yes  no  I don’t know | | **B. for yourself?**  yes  no  I don’t know | |
|  | **Can you please**  **comment?** | | | |
| **E28** | **Which of the following terms do you feel were most applicable at the time of death of your relative?**  *(you can tick one or more answers)* | | | |
|  | completely unexpected | in time | | expected |
|  | finally (after long waking) | still sudden | | suddenly |
|  | still fast | unprepared | | long expected |
|  | finally (after a long fight) | too fast | | unexpected |
|  | finally (after long suffering) | appropriate | | I don’t know |
|  | other, nl.: | | | |
| **E29** | **Which of the following terms do you feel were most applicable to the way your relative died?**  *( you can tick one or more answers )* | | | |
|  | quiet | good | | sad |
|  | hectic | shocking | | painful |
|  | panicky | intimate | | beautiful |
|  | degrading | traumatic | | moving |
|  | appropriate | restless | | peaceful |
|  | dignified | I don’t know | |  |
|  | other, nl.: | | | |
| **E30** | **How would you evaluate the quality of life during the last 3 days of life of your relative?**  Very bad            Almost perfect  0 1 2 3 4 5 6 7 8 9 10 | | | |
| **E31** | **How would you evaluate the quality of dying of your relative?**  Very bad            Almost perfect  0 1 2 3 4 5 6 7 8 9 10 | | | |

| **F** | **Bereavement**  It is, especially in the beginning, not always easy to face that someone is not coming back, and to recognize that he is no longer alive. Listed below are some comments from people like you. To what extent do these statements apply to you? Statements are about the past week. (cross on each line of the box that applies). | | | | |
| --- | --- | --- | --- | --- | --- |
|  |  | **never** | **seldom** | **sometimes** | **often** |
| **F1** | **I have the feeling that my relative is still here.** |  |  |  |  |
| **F2** | **Aloud or in my mind I talk to my relative.** |  |  |  |  |
| **F3** | **I catch myself waiting for my relative.** |  |  |  |  |
| **F4** | **Accepting the loss of my relative is very difficult for me.** |  |  |  |  |
| **F5** | **I long for my relative.** |  |  |  |  |
| **F6** | **I realize my relative is gone for good.** |  |  |  |  |
| **F7** | **There are occasions where I think to see or hear my relative.** |  |  |  |  |
| **F8** | **I feel guilty if I'm not thinking of my relative.** |  |  |  |  |
| **F9** | **I find it hard to live without my relative** |  |  |  |  |
| **F10** | **When I think of my relative it makes me sad.** |  |  |  |  |
| **F11** | **I still feel that I have not said goodbye of my relative.** |  |  |  |  |
| **F12** | **How difficult is it for you to detach yourself from thoughts and grief about your relative and to set your mind to other, perhaps new obligations?**  very difficult  somewhat difficult  not difficult | | | | |
| **F13** | **Do you receive sufficient support from family and friends in processing the loss of your relative?**  yes, more than enough  yes, enough  no, not enough | | | | |

**Space for comments and/or additions**

When did you complete this questionnaire?

**/**  **/**

day month year

You can return the questionnaire in the enclosed envelope. If you have any further questions or if you want to talk to the researcher, please contact us via the address below*.*

Mrs. F. E. Witkamp

Erasmus MC, Universitair Medisch Centrum Rotterdam

Afdeling Maatschappelijke Gezondheidszorg

Postbus 2040

3000 CA Rotterdam

e-mail: [f.witkamp@erasmusmc.nl](mailto:f.witkamp@erasmusmc.nl)

*Thank you very much for your time and your cooperation*
